# A scoping review of the feasibility, usability, and efficacy of digital interventions in older adults concerning physical activity and/or exercise

**DOI:** 10.3389/fragi.2025.1516481

**Published:** 2025-04-11

**Authors:** Ethan C. J. Berry, Nicholas F. Sculthorpe, Ashley Warner, James D. Mather, Nilihan E. M. Sanal-Hayes, Lawrence D. Hayes

**Affiliations:** ^1^ Sport and Physical Activity Research Institute, School of Health and Life Sciences, University of the West of Scotland, Glasgow, United Kingdom; ^2^ School of Health and Society, University of Salford, Salford, United Kingdom; ^3^ Lancaster Medical School, Lancaster University, Lancaster, United Kingdom

**Keywords:** ageing, exercise, physical activity, muscle strengthening, digital interventions, mHealth, eHealth

## Abstract

**Background:**

The global population is aging, leading to significant health challenges among older adults, such as reduced muscle mass, increased risks of dementias, and chronic diseases. Physical activity (PA) is crucial for maintaining health and wellbeing in this demographic, yet participation tends to decrease with age due to various barriers. Digital technologies, including mobile health (mHealth) interventions, show promise in promoting PA among older adults, though their adoption remains limited due to intrinsic and extrinsic challenges.

**Objectives:**

This scoping review aimed to systematically map existing evidence on digital PA interventions for older adults, assessing feasibility, usability, and efficacy, whilst providing recommendations for future research and practice.

**Eligibility criteria:**

Original investigations concerning digital interventions in older adults (≥60 years of age) focusing on physical activity and/or exercise were considered. Sources of evidence: Four electronic databases [MEDLINE, CINAHL Ultimate, Scopus and Cochrane Central Register of Controlled Trials (CENTRAL)] were searched.

**Methods:**

A scoping review was conducted using the scoping review methodological framework. Review selection and characterisation were carried out by two independent reviewers.

**Results:**

The 34 included studies were published between 2005 and 2023 across Europe, North America, Asia, and Oceania. Participants varied from healthy to frail individuals, with some diagnosed with dementia or cognitive impairment. Interventions were most commonly delivered via exergames, tablet apps, and videoconferencing. The most common exercise program type was multicomponent. Most studies assessed efficacy, feasibility, and usability, with many using a combination of these measures. Reminders were commonly utilised to enhance engagement through various digital and non-digital methods.

**Conclusion:**

There was a notable lack of mobile health (mHealth) studies in the literature, with most research focusing on exergame and tablet interventions. More research on smartphone apps, particularly for muscle strengthening, is needed, and the growing ease of app development may drive innovation and research. Digital interventions are generally feasible, usable, and effective for older adults, offering a promising, scalable approach for promoting PA. This review identified several valuable lessons from the existent literature for future developments.

## Introduction

### Rationale

Ageing is ubiquitous amongst humans and in recent years the global population aged rapidly (Lu et al., 2023). In 2018, the over 65s outnumbered children under 5 years of age for the first time in history and it is expected that by 2050, 22% of the global population will be over 65 (Lu et al., 2023). With ageing comes several health challenges such as loss of muscle mass, increased risks of dementia and cognitive impairment, elevated blood pressure, heart disease, and diabetes mellitus (Wilson et al., 2021), all of which have significant impact on older adults’ abilities to complete activities of daily living. Consequently, health and wellbeing has become a priority, evidenced by the United Nations Sustainable Development Group’s (UNSDG) goals, particularly healthy lives and wellbeing at all ages (UNSDGD 3) (Nations, 2022).

One key strategy that has become apparent for maintaining health and wellbeing for older adults is physical activity (PA) and/or exercise (Graham et al., 2024). Exercise and/or PA has been shown to exert a range of physical and mental benefits (Hayes et al., 2020; Sciamanna et al., 2021). PA refers to any bodily movement produced by skeletal muscle that requires energy expenditure, while exercise is a subcategory of PA that follows a plan and structure with repetition (Taylor et al., 2021) (exercise/PA will be referred to as PA throughout this review). Despite the benefits of PA, as people age, they typically become more sedentary (Pollet et al., 2020). This reduction can be attributed to the unique challenges older adults face as a consequence of ageing such as decreased mobility, chronic health conditions, and social isolation (Taylor et al., 2021).

The emergence of digital technology has shown promise for promoting PA in older populations (Langhammer et al., 2018). One digital intervention type which has shown potential is mobile health (mHealth; mostly using mobile apps). This refers to the practice of medicine and public health supported through mobile devices (Istepanian, 2022). Similarly, electronic health (eHealth) refers to the practice of medicine and public health supported through digital technologies such as tablet computers, computers, and laptops (Da Fonseca et al., 2021).

Around 90% of older adults own a laptop or computer and, in the United Kingdom, approximately 70% of people over 60 years of age own a smartphone [around 67% worldwide (Changizi and Kaveh, 2017)], suggesting older adults are more digitally literate and connected than ever before (Changizi and Kaveh, 2017). The large number of older adults with eHealth and mHealth access now makes technology-enabled PA interventions possible. Although features such as push notifications, daily reminders, support, and feedback are possible with traditional technology interventions, accessibility and scalability are enhanced when mHealth is deployed (Stockwell et al., 2019). Interventions utilising smartphone applications (apps), wearables, exergames, and web platforms have been used in recent years (Langhammer et al., 2018). One benefit of using eHealth for PA interventions is it enhances the acceptability, efficacy, and sustainability of PA interventions for older adults (Cunningham et al., 2020).

Despite the potential for digital interventions to promote PA in older adults, their adoption in this population remains low compared to others (Pywell et al., 2020). Specific factors which are relevant to these age groups may indicate why interventions of this nature have either not been adopted or adopted poorly. A Previous review has identified various intrinsic factors such as memory, hearing, motor control, and feelings of incompetence (Lu et al., 2023) as some of the intrinsic factors affecting adherence. Extrinsic factors such as cultural barriers, the belief that smartphones are for phone calls only, lack of digital literacy and privacy and security concerns surrounding technology use, are some of the extrinsic barriers to participation (Lu et al., 2023). By involving older adults in the design process, addressing their specific needs, and continuously evaluating these criteria, digital interventions can become more effective and widely adopted in promoting PA among older adults. By not addressing these barriers it is possible that digital technology as a means of encouraging PA will not meet its full potential (Pywell et al., 2020). To address this, it is essential to evaluate the feasibility, usability, and efficacy of these interventions. The feasibility of such studies depends on older adults’ willingness to participate, which can be influenced by their familiarity with technology and their perceived ease of use (Loh et al., 2018). Usability involves assessing how user-friendly and accessible these digital interventions are for older adults, considering their specific needs and limitations (Loh et al., 2018). Efficacy measures how effective these interventions are in increasing PA levels and improving health outcomes (Shah et al., 2021). Criteria for determining feasibility include recruitment rates, retention rates, and participants’ ability to navigate and use the technology (Teresi et al., 2021). Usability can be evaluated through user satisfaction, task completion rates, and the frequency of technical issues encountered (Lyon et al., 2020). Efficacy is determined by measuring changes to PA levels, fitness improvements, and other health metrics pre- and post-intervention (Cunha and Gonçalves, 2015).

### Objectives

Considering the challenges and opportunities discussed, we thought it was pertinent to map the existing evidence on digital interventions concerning PA in older adults. A scoping review can systematically map the literature to identify paucities and limitations, and generate insights for future research, practice, and policy (Campbell et al., 2023). This review will assess the feasibility, usability, and efficacy of these interventions in older adults. By examining digital interventions for PA, we aim to highlight successful and unsuccessful strategies, informing the development of digital interventions for PA in older adults. Additionally, we will compare various digital intervention approaches to encourage the integration of diverse strategies in future research. Focusing on specific domains of PA (e.g., muscle strengthening, aerobic conditioning) will enhance our understanding of whether digital interventions support older adults. Our specific objectives for this scoping review were to (1) conduct a systematic search of the literature on digital interventions in relation to PA in older adults, (2) map the types and characteristics of the digital interventions used (mobile apps, tablet devices, wearables), (3) outline outcomes reported in each intervention (usability, feasibility, and efficacy), (4) understand user perspectives (preferences, feedback) – focusing on experiences, needs and challenges, with a view to inform future mHealth approaches for older adults, and (4) provide recommendations for advancement in the area.

## Methods

### Protocol and registration

The review was completed in accordance with the Arskey and O’Malley (Arksey and O’Malley, 2005) methodological framework, which does not include pre-registration. The review adhered to the guidelines outlined in the Preferred Reporting Items for Systematic Reviews and Meta-Analyses Extension for Scoping Reviews (Tricco et al., 2018), during both its execution and reporting (Tricco et al., 2018).

### Eligibility criteria

Studies were included in our review if they met the following inclusion criteria: [1] Human participants ≥60 years of age which is deemed the start of old age by the United Nations (United Nations, 2024) and has been applied in previous, similar reviews (Wilson et al., 2021; Hayes et al., 2020), [2] Human participants living independently in the community, [3] Published in English, [4] Digital interventions relating to the implementation of apps, wearable technology, tablets, smartphones, web calls, and web apps which aim to improve adherence, uptake, acceptance, or outcomes of PA and [5] Includes outcome measures on feasibility, usability, or efficacy for the digital intervention. Papers were not included if [1] They were not published in English language, [2] They had human participants with a mean of <60 years of age, [3] They were review papers, [4] They were abstracts, conference papers, or protocols, [5] They did not involve PA, [6] They not use a digital intervention, [7] They did not include outcome measures on feasibility, usability, and efficacy for the digital intervention, [8] They included other variables of interest over and above PA and [9] They did not take place in the community. We included studies which included participants with comorbidities, as ageing is associated with multimorbidity (Marengoni et al., 2011).

### Search strategy

The search strategy for the review consisted of a combination of keyword and MeSH term searching. The following search was applied in the MEDLINE database: (communit* N3 dwell* or residen*) AND (elderly or geriatric or age* or aging) AND {text* or SMS or “mobile device” or “mobile phone” or “mobile health” or mHealth or eHealth or internet-based or web-based or DVD-based or [wearable N3 (devic* OR technol*)] or computer or “computer assisted” or (serious N3 game*) or tablet or “artificial intelligence” or AI}. We chose to omit searching for outcomes directly, as recommended previously (Frandsen et al., 2022), due to the broad scope of possible outcomes relating to PA or feasibility usability and efficacy of interventions. We utilised filters when searching within databases to ensure only studies published in English with human participants appeared in our search. The full search protocol can be found in Supplementary Material 1.

### Information sources

Four electronic databases (MEDLINE, CINAHL Ultimate, Scopus and the Cochrane Central Register of Controlled Trials (CENTRAL) were searched to identify original research articles published from 1 January 1995, to 11 January 2024. We chose 1995 because this was the time of initial commercialisation of the internet, paving the way for web interventions (Naughton, 2016). After this, mobile and wearable technology was developed and implemented in exercise settings (Pagliari et al., 2005). By searching within this time frame, the full spectrum of internet-enabled digital interventions would be captured. Citation mining was also conducted for eligible papers.

### Study selection

Once the scoping search was completed, all records were then downloaded into a single reference list using Zotero (version 6.0.26) and duplicates were removed using the de-duplication function. From there, records were uploaded to Rayyan (Ouzzani et al., 2016) software for screening. Firstly, titles and abstracts were screened by the first author (EB) utilising the include/exclude/maybe and labelling functions in Rayyan in line with the inclusion/exclusion criteria. This was then confirmed by second author (JM) and agreement was reported via Cohen’s kappa statistic. Regular collaborator meetings were scheduled, where conflicts were discussed and resolved. These involved members of the research team explaining their reasons for including/excluding a study. Once titles and abstracts were reviewed, the included studies full texts were sourced and read in full by the first author (EB) in line with the inclusion/exclusion criteria, this was then confirmed by second author (JM) and agreement was reported. Conflicts were again resolved during reviewer meetings, and if they could not be resolved, a third reviewer (LH) decided the inclusion or exclusion of an article.

### Data extraction

Data extraction was completed by the first author (EB) using a pre-built Microsoft Excel (version 16.79.3) table. Data extracted included author(s), geographical location, study design and aim(s)/objective(s), N of participants, participant characteristics, digital intervention description, PA domain frequency of reminders, study setting, reported outcomes, adherence/compliance/attendance, and key findings. Considering the varied methodologies and outcomes our search elicited we tabulated the results into a data extraction table to allow for a narrative synthesis.

### Outcome measures

The main outcomes reported in each study were measures of feasibility, usability, and efficacy. In terms of feasibility, we anticipated measures on recruitment rate, retention rate, adherence, cost effectiveness and logistical challenges. In terms of usability, we expected measures on ease of use, user satisfaction, learnability, error rate, and task efficiency. For efficacy we expected measures on clinical outcomes, functional outcomes, behavioural outcomes, and quality of life.

## Results

### Study selection

**FIGURE 1:**
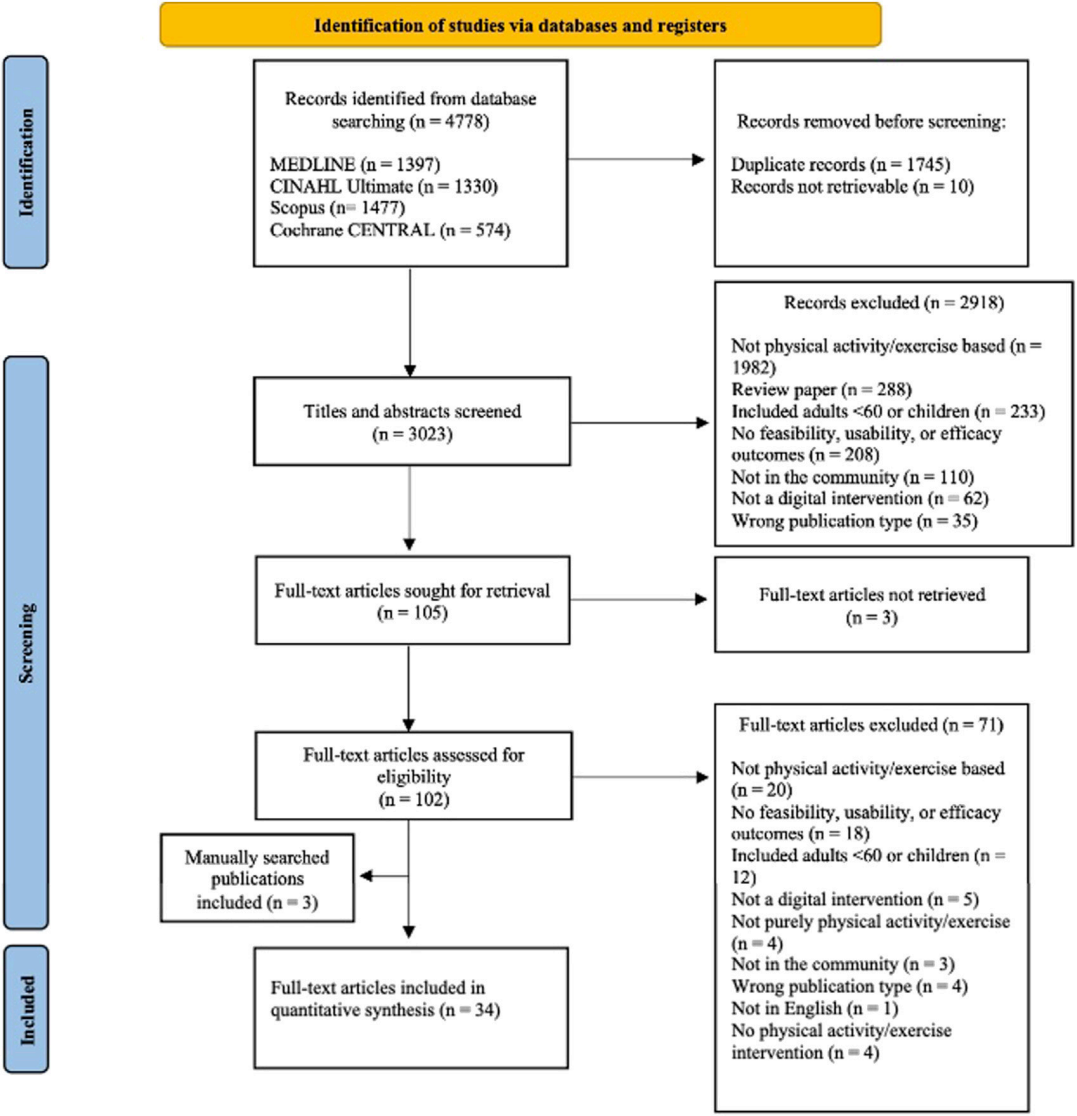
Records identified through database and reference list searching.

Following the initial database search, 4,778 articles were identified (Figure 1) and 3,023 titles and abstracts were screened once duplicates were removed (k = 1745). Ten articles were not retrievable from databases. This resulted in 2,918 articles being removed in line with the inclusion criteria and 102 full text articles being screened for eligibility as three full texts were not retrievable. Of these 102, 71 were removed, leaving 31 articles, a further three articles were identified by searching the reference lists of included articles and therefore a final total of 34 articles were included in the review. At the titles and abstract stage blind agreement between reviewers indicated via the Cohen’s Kappa statistic was 0.95 indicating almost perfect agreement and at the full text stage this was 0.39 indicating fair agreement.

### Study characteristics

**FIGURE 2:**
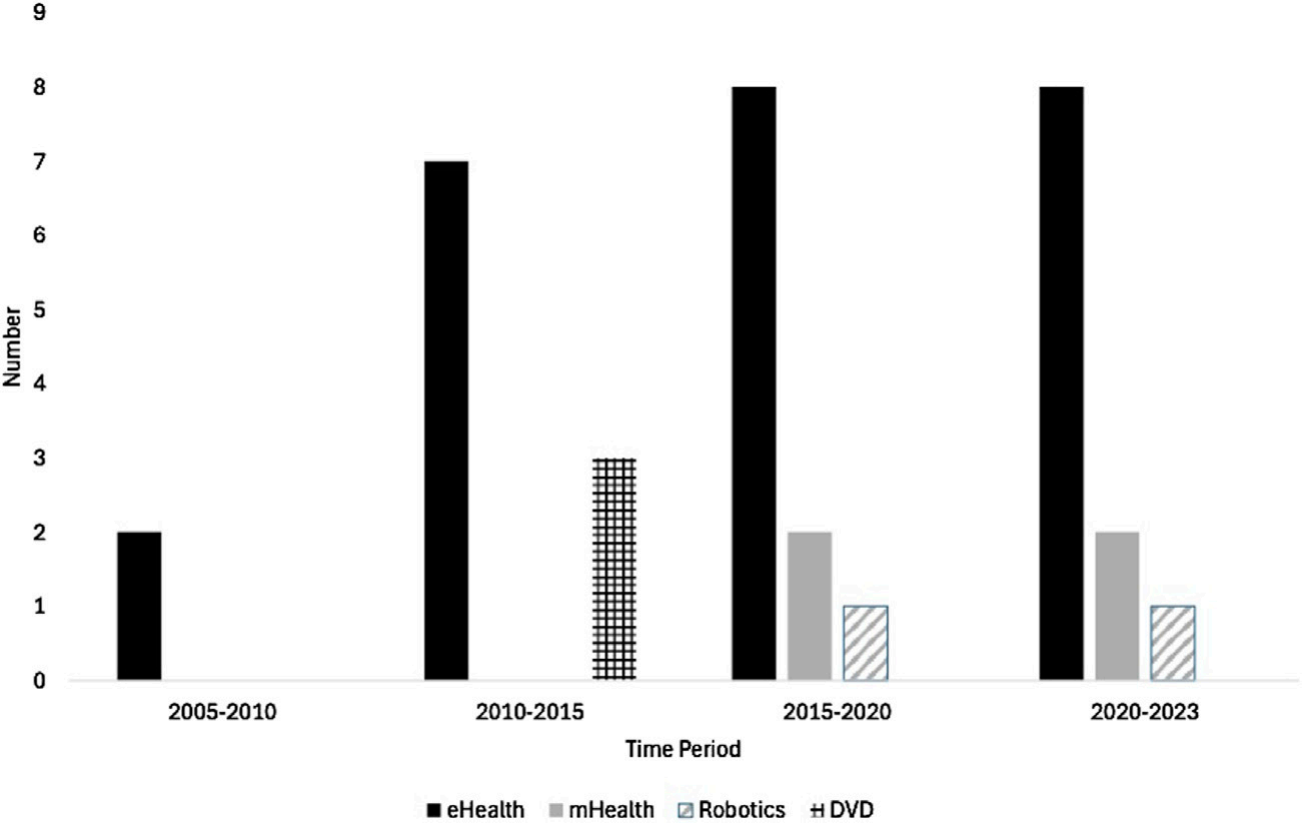
Distribution of digital intervention types from 2005–2023.

Of the 34 studies included, publication year range spanned from 2005–2023 (Figure 2). Intervention locations spanned Europe, North America, Asia, and Oceania. As shown in Supplementary Table 1, 13 of the studies were randomised controlled trials (RCTs) (Bowen et al., 2022; Gothe et al., 2014; Liu et al., 2021; Karssemeijer et al., 2019; Lee et al., 2022; Szturm et al., 2011; Yang et al., 2020; Yamada et al., 2011; Montero-Alía et al., 2019; Gschwind et al., 2015; Padala et al., 2017; Delbaere et al., 2021; Alley et al., 2023), nine were feasibility studies (Mair et al., 2022; Schwartz et al., 2021; Taylor et al., 2019; Mansson et al., 2020; Silveira et al., 2013; Li et al., 2022; Wong et al., 2005; Granet et al., 2023; Jansons et al., 2021; Li et al., 2020), three were randomised intervention trials (Granet et al., 2022; Shake et al., 2018; Franco et al., 2012), three were pre-test/post-test designs (Bieryla and Dold, 2013; Katrancha et al., 2015; Roopchand-Martin et al., 2015), two were pilot studies (Daly et al., 2021; Rosenberg et al., 2010), one was a crossover trial (Ozaki et al., 2017), one was a preclinical exploratory trial (van Het Reve et al., 2014) and one was a prospective cohort study (Geraedts et al., 2017). All studies reported sample size and included community dwelling older adults (>60). Participants were a mixture of healthy, inactive, or frail individuals and others included people diagnosed with dementia or cognitive impairment. The most popular digital intervention mode (k = 10) was exergames carried out at home or at senior community centres, eight used tablet-based approaches, five used videoconferencing mainly via Zoom, three used DVDs, three used a combined wearable and smartphone intervention, two used robotics, one used a combined wearable and tablet intervention, one used a smartphone intervention via an application with the option to also download onto a tablet, and one used an Amazon Alexa voice activated device (similar to a tablet intervention as this was delivered through an app on a touch screen version of the Alexa).

**TABLE 1:** Summary of the key findings pertaining to efficacy of digital interventions in relation to physical performance metrics.

Study	Study duration	Intervention	Participant N (mean ± SD Age)	Sex	Outcome measure	% Increase/Decrease from baseline	Sig
Granet et al. (2022)	12 weeks	Videoconferencing intervention conducted via Zoom. Mixture of aerobic, functional and resistance training 3x 1-h sessions per week	83 participants (70 ± 5.1)	M = 16 F = 67	SPPB 10STS 30-s CST	Live group = 5% Recorded group = −1% Live group = 60% Recorded group = 10% Live group = 33% Recorded group = 22%	Yes No Yes No Yes No
van Het Reve et al. (2014)	12 weeks	Tablet intervention conducted via the “ActiveLifestyle” app. Mixture of 2x resistance and 5x balance training sessions per week	44 participants (75 ± 8.6)	M = 16 F = 28	SPPB	Brochure group = 12% Social group = 23% Individual group = 11%	Yes Yes Yes
Bieryla and Dold (2013)	3 weeks	Exergame intervention conducted via the Wii Fit. Mixture of 3x sessions of balance and aerobic sessions per week	12 participants (82 ± 5.5)	M = 2 F = 10	BBS FAB FRT TUG	Experimental = 6% Control = 5.8% Experimental = 5% Control = 3% Experimental = −3% Control = 0% Experimental = −6% Control = −12.5%	Yes No No No No No No No
Karssemeijer et al. (2019)	12 weeks	Exergame intervention conducted via an exercise bike connected to a screen. Aerobic training 3x per week for 30–50 min at 65%–75% HR reserve	115 participants (79 ± 6.9)	M = 62 F = 53	TUG 5TSTS 10 m walk test SPPB	Experimental = - 2% Control = 4% Experimental = −8% Control = 7% Experimental = - 4% Control = −8% Experimental = 4% Control = 2%	No No No No No No No No
Lee et al. (2022)	12 weeks	Robotics intervention delivered via hip exoskeleton. Mixture of weekly walking and resistance activity dependent on study group	60 participants (75 ± 4.1)	M = 30 F = 30	10 m walk test SPPB BBS FRT TUG	Largest increase reported per test by group Group D = 7% Group D = 7% Group D = 8% Group D = 18% Group B = −21%	Yes Yes Yes Yes
Szturm et al. (2011)	8 weeks	Exergame intervention. Strength training completed 2x per week for 45 min	30 participants (81 ± 6.5)	M = 11 F = 19	BBS TUG	Experimental = 21% Control = 21% Experimental = 20% Control = 51%	Yes Yes Yes Yes
Katrancha et al. (2015)	12 weeks	DVD intervention. Aerobic and balance training completed 3x per week for 45 min	32 participants (73 ± 8.6)	M = 3 F = 29	COB measured via the Wii Fit balance board	Eyes open right = 3% Eyes open left = −3%	Yes Yes
Yang et al. (2020)	5 weeks	Exergame intervention. Balance training completed 2x per week for 45 min	20 participants (68)	M = 2 F = 18	30 Sec-CST TUG FRT OLST	Experimental = 38% Control = 21% Experimental = −14% Control = −13% Experimental = 16% Control = 18% Experimental = 146% Control = 17%	Yes Yes Yes No Yes Yes Yes Yes
Shake et al. (2018)	10 weeks	Tablet intervention delivered via the ‘Bingocize’ app. Mixture of aerobic, balance and resistance training completed 2x per week for 1 h	105 participants (73 ± 7.8)	M = 15 F = 90	30-s CST 4 m walk test	Experimental = - 17% Control = −5% Experimental = 8% Control = 6%	Yes No No No
Yamada et al. (2011)	24 weeks	DVD intervention. Resistance and agility training completed 2x per week for 20 min	84 participants (83 ± 6.1)	M = 19 F = 65	TUG 5TSTS	Experimental = 2% Control = −2% Experimental = −2% Control = - 1%	No No No No
Montero-Alía et al. (2019)	12 weeks	Exergame intervention delivered via the Wii Fit. Balance training completed 2x per week for 30 min	977 participants (75)	M = 400 F = 577	Tinetti’s Balance Test	Experimental = 0% Control = 2%	No No
Roopchand-Martin et al. (2015)	6 weeks	Exergame intervention delivered via the Wii Fit	33 participants (70 ± 6.7)	M = 7 F = 26	BBS	Single group pre-test/post-test = 3%	Yes
Wong et al. (2005)	12 weeks	Videoconferencing intervention. Resistance and functional training completed 3x per week	20 participants (75 ± 7)	M = 2 F = 18	TUG BBS	Single group pre-test/post-test = −21% Single group pre-test/post-test = 12%	Yes Yes
Franco et al. (2012)	3 weeks	Exergame intervention delivered via the Wii Fit. Balance training completed 2x per week 10–15 min	32 participants (78 ± 6)	M = 7 F = 25	BBS Tinetti’s balance test	Wii Fit = 7% MOB = 7% Control = 2% Wii Fit = 3% MOB = 5% Control = 4%	No No No No No No
Granet et al. (2023)	12 weeks	Videoconferencing intervention delivered via Zoom. Mixture of aerobic and resistance training completed 3x per week for 1 h	46 participants (60)	M = 13 F = 33	SPPB TUG 30-s CST	Live-recorded-live group = 7% Recorded-live-recorded group = 2% Live-recorded-live group = - 8% Recorded-live-recorded group = - 8% Live-recorded-live group = 31% Recorded-live-recorded group = 30%	No No No No Yes Yes
Gschwind et al. (2015)	12 weeks	Exergame intervention delivered via Microsoft Kinect. Balance training completed 3x per week for 40 min and resistance training completed 3x per week for 15–20 min	153 participants (75 ± 6.5)	M = 60 F = 93	SPPB TUG	Experimental = 8% Control = 7% Experimental = - 2% Control = - 10%	No No No No
Padala et al. (2017)	8 weeks	Exergame intervention delivered via the Wii Fit. Aerobic and resistance training completed 3x per week for 45 min	30 participants (68 ± 6.7)	M = 26 F = 4	BBS	Experimental = 8% Control = 0%	Yes No
Jansons et al. (2021)	12 weeks	Voice activation intervention delivered via Amazon Alexa. Resistance training completed in ‘snacks’ at 2x per day, 3x per day and 4x per progressing in 4-week stages	15 participants (70 ± 4)	M = 6 F = 9	30-s CST	Single group pre-test/post-test = 10%	No
Delbaere et al. (2021)	2 years	Tablet intervention delivered via the ‘StandingTall’ app. Balance training completed 2 h per week minimum	503 participants (77 ± 5.5)	M = 164 F = 339	TUG 5TSTS 10 m walk SPPB	Experimental = −3% Control = 0% Experimental = - 11% Control = −7% Experimental = - 2% Control = - 2% Experimental = 0% Control = 0%	No No No No No No No No
Ozaki et al. (2017)	12 weeks	Robotics intervention delivered via the ‘BEAR’ system. Resistance and balance training completed 2x per week	27 participants (73 ± 6)	M = 7 F = 20	Gait speed TUG FRT	Experimental = 4% Control = 2% Experimental = - 7% Control = −3% Experimental = 10% Control = 1%	Yes No Yes No Yes No

The outcome measures described in the table are as follows; Short Physical Performance Battery (SPPB), Berg Balance Scale (BBS), Timed Up and Go (TUG), Centre of Balance Dispersion (COBD), 30 s chair stand test (30-s CST), One Leg Stand Test (OLST), Fullerton Advanced Balance Test (FAB), Functional Reach Test (FRT), 10 sit to stands (10STS).

A total of 18 studies employed a multicomponent PA intervention (aerobic, resistance, balance, and flexibility exercise), six used only balance training, four used only aerobic training, three used resistance training and two used step goals. A total of 18 studies employed reminders that were either built into the digital technology (calendar reminders or push notifications) or delivered via phone calls, text messages, emails, or home visits. In total, 23 studies reported outcome measures on efficacy, 19 reported feasibility and 11 reported usability. Some studies used a mixture of these outcomes.

#### Feasibility

Of the 20 studies that evaluated the feasibility of the digital intervention used (Bowen et al., 2022; Liu et al., 2021; Karssemeijer et al., 2019; Yang et al., 2020; Gschwind et al., 2015; Padala et al., 2017; Mair et al., 2022; Schwartz et al., 2021; Taylor et al., 2019; Mansson et al., 2020; Silveira et al., 2013; Li et al., 2022; Wong et al., 2005; Granet et al., 2023; Jansons et al., 2021; Granet et al., 2022; Daly et al., 2021; Rosenberg et al., 2010; van Het Reve et al., 2014; Geraedts et al., 2017), 19 (out of 20; 95%) concluded the intervention was feasible in older adults. Digital interventions were feasible when delivered via videoconferencing on the Zoom platform (Li et al., 2022; Wong et al., 2005; Granet et al., 2023; Granet et al., 2022; Rosenberg et al., 2010), exergames (Karssemeijer et al., 2019; Yang et al., 2020; Gschwind et al., 2015; Padala et al., 2017; Rosenberg et al., 2010), tablets and voice activation (Taylor et al., 2019; Silveira et al., 2013; Jansons et al., 2021; Daly et al., 2021; van Het Reve et al., 2014), smartphones combined with wearables (Bowen et al., 2022; Liu et al., 2021; Mair et al., 2022) and smartphones used independently (Mansson et al., 2020). Geraedts et al. (2017) reported that a 6-month intervention combining a wearable activity necklace and tablet app was not feasible as they did not meet their adherence target (69%) because of internet connection issues.

Among the studies evaluating feasibility, definitions varied. Some considered feasibility as the proportion of sessions completed or adherence to the intervention. Others defined it by participant satisfaction rates or dropout rates. Additionally, some studies focused on adverse events, defined as intervention-related incidents causing injury or study absence. Further definitions included the ability to recruit participants to target or the efficiency of technical and operational aspects. Many studies combined these criteria to assess the feasibility of their interventions. Feasibility metrics varied from study to study with a total of seven different measures used, with some using a combination of measures. Of the 20 studies 16 (80%) reported adherence which was based on a percentage calculation at the end of the study (Liu et al., 2021; Karssemeijer et al., 2019; Yang et al., 2020; Mair et al., 2022; Schwartz et al., 2021; Taylor et al., 2019; Mansson et al., 2020; Silveira et al., 2013; Wong et al., 2005; Granet et al., 2023; Jansons et al., 2021; Granet et al., 2022; Daly et al., 2021; Rosenberg et al., 2010; van Het Reve et al., 2014; Geraedts et al., 2017), ten (47%) used participant satisfaction surveys, questionnaires and user evaluations, five (26%) measured the percentage of adverse events (Bowen et al., 2022; Liu et al., 2021; Padala et al., 2017; Schwartz et al., 2021; Taylor et al., 2019; Mansson et al., 2020; Silveira et al., 2013; Li et al., 2022; Granet et al., 2023; Jansons et al., 2021; Li et al., 2020; Daly et al., 2021; Geraedts et al., 2017), four (21%) measured attrition (drop-out) rate (Liu et al., 2021; Mansson et al., 2020; Silveira et al., 2013; Li et al., 2022), three (16%) calculated the retention rate (Li et al., 2022; Jansons et al., 2021; Daly et al., 2021), and two (11%) measured the recruitment (Silveira et al., 2013; Li et al., 2022).

### Adherence

Of the studies reporting adherence (16), information was given on how many sessions completed, attended or interactions with the technology. Of the 16 studies measuring adherence 14 (85%) reported high adherence levels in their intervention ranging from 54% to 115% with many applying a minimum criterion. Two studies did not meet their adherence criteria when participants were asked to wear a wearable activity necklace synced with a tablet app (Geraedts et al., 2017) and when participants were given an at home exergames intervention set up via their home television (Gschwind et al., 2015).

### Questionnaires, surveys and interviews

A total of 11 studies employed the use of questionnaires or surveys. Nine of the 11 studies reported users to be satisfied with the intervention and happy with their experience. One study which used a wearable activity necklace synced with a tablet app (Geraedts et al., 2017) reported that some participants were unsatisfied with being left to do PA remotely and would prefer the research team to be in regular contact, they also stated they found the intervention hard to participate in due to internet connection issues. A further study reported that participants flagged technical issues (Li et al., 2020).

#### Adverse events

None of the included studies reported serious adverse events during their digital interventions however, two studies reported minor events (Taylor et al., 2019; Daly et al., 2021). One of these adverse events involved an incident where a participant fell while completing PA via a tablet app, no injury was sustained. The other adverse event involved a participant sustaining a strained calf during completing PA via a tablet app, no further injury was sustained.

### Attrition, recruitment and retention

10 out of the included studies reported on attrition, recruitment, and retention for their digital intervention. One study reported 17% attrition in their smartphone intervention group which was less than the non-digital intervention group (Mansson et al., 2020). A further study employing a tablet app reported a 17% attrition rate but only 7% recruitment rate (Silveira et al., 2013) and another study using a tablet app reported a 95% retention rate (Daly et al., 2021). One investigation employing a smartphone app alongside a wearable had a recruitment rate of 93% and attrition rate of 0% (Liu et al., 2021). One study which used videoconferencing via Zoom had an 11% attrition rate and 94% retention rate (Li et al., 2022).

#### Usability

Of the 12 studies which evaluated usability of their digital intervention five used a questionnaire/survey/enjoyment measuring approach (Alley et al., 2023; Li et al., 2022; Granet et al., 2023; Rosenberg et al., 2010; Ozaki et al., 2017), four used the system usability scale (SUS) (Alley et al., 2023; Taylor et al., 2019; Jansons et al., 2021; Daly et al., 2021), one measured interaction with their digital app (Silveira et al., 2013), one used a technical and operational survey (Geraedts et al., 2017), and one used an interview (Liu et al., 2021). Of the 11 studies reporting outcomes on usability all (100%) reported positive usability findings for combined wearable and smartphone interventions, tablet apps, exergames, videoconferencing, smartphone apps, combined wearable and tablet interventions and robotics.

### Questionnaires and enjoyment scales

Of the six studies measuring usability via questionnaires or enjoyment scales five of these studies reported positive feedback from participants regarding the usability of the digital intervention. Alley et al. (2023) reported in a combined tablet app and wearable intervention that only 44% of participants found the in-built planning tool usable and only 51% found the PA plans usable.

### System usability scale

Three of four studies employing the SUS garnered positive results. Taylor et al. (2019) had a mean SUS rating of 68 for a tablet app intervention, Jansons et al. (2021) had a mean rating of 75 for a voice activated intervention, and Daly et al. (2021) had a mean rating of 86 for a tablet intervention, all of which are deemed above average usability. In the study by Alley et al. (2023) the mean SUS score was 61, which is below average.

### Interactions with technology and technical and operational usability

One study reporting usability via interaction with the technology during the intervention reported positive results for a balance and strength intervention delivered via a tablet app (Silveira et al., 2013). It was reported 91% of participants could navigate messages posted on the apps in-built bulletin board and 100% could read the messages. However, the writing activities were not as usable as 64% were not able to write on the bulletin board and 46% were not able to write on the public inbox. One study reported technical and operational usability (Geraedts et al., 2017) and reported issues with connection and navigation of the app (29 incidents).

### Interviews

The single study employing an interview approach for a wearable and smartphone intervention revealed that 20 out of 21 participants agreed the wearable was easy to use and 80% agreed the app was easy to use. Some participants stated that they did not fully utilise the app but may have done so if it included more features.

#### Efficacy

Of the 23 studies reporting outcomes on efficacy (Bowen et al., 2022; Gothe et al., 2014; Karssemeijer et al., 2019; Lee et al., 2022; Szturm et al., 2011; Yang et al., 2020; Yamada et al., 2011; Montero-Alía et al., 2019; Gschwind et al., 2015; Padala et al., 2017; Delbaere et al., 2021; Li et al., 2022; Wong et al., 2005; Granet et al., 2023; Jansons et al., 2021; Granet et al., 2022; Shake et al., 2018; Franco et al., 2012; Bieryla and Dold, 2013; Katrancha et al., 2015; Roopchand-Martin et al., 2015; Ozaki et al., 2017; van Het Reve et al., 2014), only five completed an *a priori* sample size calculation (Yamada et al., 2011; Shake et al., 2018). A range of tools were used to report on this including physical health measures, muscular power, physical performance measures, muscular endurance, PA, balance testing, muscular strength, cognition, and questionnaires.

### Physical performance

In total, 20 studies reported on measures of physical performance (Karssemeijer et al., 2019; Lee et al., 2022; Szturm et al., 2011; Yang et al., 2020; Yamada et al., 2011; Montero-Alía et al., 2019; Gschwind et al., 2015; Padala et al., 2017; Delbaere et al., 2021; Wong et al., 2005; Granet et al., 2023; Jansons et al., 2021; Granet et al., 2022; Shake et al., 2018; Franco et al., 2012; Bieryla and Dold, 2013; Katrancha et al., 2015; Roopchand-Martin et al., 2015; Ozaki et al., 2017; van Het Reve et al., 2014). Of these studies, three reported significant increases in SPPB from baseline in their experimental group ranging between 5% and 12%, with the largest increase to a mean score of 12, deemed high. One study reporting on 10STS reported a significant increase of 60%, this increase was calculated via an index score and essentially meant participants were able to complete 10STS repetitions quicker post intervention. Of the five studies reporting on the 30-s CST, four reported significant increase from baseline ranging between 30% and 38%, the study with the largest increase was able to increase 30-s CST repetitions by 5.5–20, which meets healthy criteria for the age group. Of the six studies reporting on the BBS, five reported significant increases from baseline ranging between 6% and 21%, the study with the largest increase had participants with scores in the 40s post intervention, indicative of being able to safely walk without assistance. Of the four studies reporting FRT, three reported significant increases from baseline between 10% and 18%, the study with the largest increase had participants increase to an FRT value of 26cm, which is normative for their age group. Of the 11 studies reporting on TUG, five reported significant reductions in TUG time from baseline ranging between 7% and 51%, resulting in participants being able to complete this in under 15 s, which is still below average for the age group. The sole study reporting on COB measured via the Wii Fit balance board reported a significant improvement of 3%. The one study reporting on the OLST reported significant improvements of 146% and 17% in the experimental and control group respectively, meaning the experimental group could stand on one leg for 12 s longer post intervention, bringing them, in line with reference values for their age. The singular study reporting on gait speed reported a significant improvement in the experimental group of up to 4% from baseline, making the intervention group 3 m/min faster post intervention.

### Muscular power, endurance, and strength

Of the 23 studies, six reported on either muscular power, endurance, or strength. Granet et al. (2022) used a videoconferencing intervention to improve muscular function and reported improvements of 21.4 in muscle power index score and 5 more sit to stand completions in the live group. Lee et al. (2022) reported improvement in lower extremity muscle strength for all three groups in their wearable robotic intervention measured via a digital dynamometer. Karssemeijer et al. (2019) reported no improvements in muscular strength or endurance measured via the five times sit to stand test after a 12-week exergame intervention. Wong et al. (2005) reported significant improvements in quadriceps strength after a 12-week videoconferencing intervention. Ozaki et al. (2017) reported improvement in lower extremity muscle strength for the intervention group compared to controls after a 12-week robotics intervention targeting muscle strength and balance. Shake et al. (2018) reported significant strength improvements in the arm curls test of up to 28%.

### Physical activity

Of the 23 studies, three reported on PA levels. Gothe et al. (2014) reported an up to 7 min per week, improvement in objectively measured PA post 6-month DVD intervention. In contrast, Karssemeijer et al. (2019) found no significant improvement in PA measured via the PA scale for the elderly (PASE) after a 12-week exergame intervention. Li et al. (2020) reported that PA measured via a Moto 360 smartwatch was increased by 41.5 counts/minute in a 6-week wearable and tablet app intervention.

### Questionnaires

Of the 23 studies, three reported on efficacy via questionnaires. Gothe et al. (2014) reported a positive treatment effect seen through the Godin Leisure Time Exercise Questionnaire (GLTEQ). Further to this Wong et al. (2005) observed improvements in the short form health survey (SF-36) score. Jansons et al. (2021) saw positive changes in EQ-5D (a standardised measure of health-related quality of life) score after their 12-week voice activated intervention.

### Physical health measures

Of the 23 studies reporting on efficacy of the digital intervention, one study reported physical health measures. Bowen et al. (2022) showed a reduction of 2.2 inches in waist circumference and 2.5lbs loss in weight compared to the control group in a wearable and smartphone combined intervention.

## Discussion

### Principal findings

The review summarises existing literature, highlighting strengths, limitations, and key issues to guide future research opportunities. Our first objective was to conduct a systematic search of the literature on digital interventions in relation to PA in older adults. An in-depth search of the current literature was completed, and 34 studies were identified. Studies included in this review used a range of digital interventions including exergames, tablet-based apps, videoconferencing, DVDs, smartphone interventions, combined wearable and smartphone/tablet interventions and robotics.

### Intervention delivery

The types and characteristics of the digital interventions reported in this review were exergames (k = 10), tablet apps (k = 9), videoconferencing (k = 5), DVDs (k = 3), combined wearable and smartphone interventions (k = 3), combined wearable and tablet interventions (k = 1), robotics (k = 2) and smartphone only interventions (k = 1). This may be surprising as ownership of smartphones far outstrips exergame ownership, tablet ownership, DVD player ownership, and wearable ownership (Rosales and Fernández-Ardèvol, 2019). However, it is important to consider timelines as the present review included studies spanning from 2005–2023. Before 2007, there were no software development kits (SDKs) for Apple or Android smartphones (Pybus and Coté, 2024) making it technically impossible to develop a mobile intervention. Furthermore, it may be surprising that exergames were the main intervention type included in the present review as recreational gaming is lowest in this age group (Zelinski and Reyes, 2009). However, previous literature has demonstrated that exergames as a mode of delivery are desired as they help overcome exercise barriers for older adults by introducing an element of fun while providing physical and cognitive engagement (Rytterström et al., 2024). However, only one included study by Gschwind et al. (2015) used a co-design approach consulting older adults during the design phase of their intervention, which is a key step in ensuring this intervention type can be executed effectively.

In terms of reach, mHealth would be the most pragmatic means to engage older adults. In terms of scalability, mHealth would also be superior to videoconferencing, robotics, and DVD-based interventions (Tomlinson et al., 2013), given the potentially automated nature of mHealth. Specific elements of mHealth such as real time feedback and personalisation help interventions by motivating individuals and crafting workouts based on fatigue levels (Kuru, 2023). A key strength of mHealth studies is the ability to use push notification reminders to enhance adherence to the intervention. A push notification is defined as an alert generated by an application when the app is not open which notifies the user of a new message or updates, which is particularly important in older adults due to the need for a focus on safety, motivation and reminders (Liu et al., 2023). For example, the included study by Liu et al. (2021) utilised reminders via the Fitbit app which notified participants via their mobile phones and wearable which 55% of the sample agreed increased their exercise self-efficacy. However, it could be argued some of the included mHealth studies have not used mHealth capabilities to their full potential. For example, an included study by Bowen et al. (2022) only used text message reminders. Studies like this may benefit from taking advantage of more features such as push notifications within apps to bolster the intervention delivery (Hernández-Reyes et al., 2019). While many studies have yet to fully explore the comprehensive potential of mHealth interventions, it is technically feasible to implement such systems. For instance, the included study by Mair et al. (2022) developed an mHealth intervention that successfully integrated behaviour change theory, incorporating elements such as goal setting, automated push notifications, and queries to external servers (in this case, weather services). This approach highlights the capability of mHealth platforms to achieve data fusion, effectively enhancing support for physical activity interventions.

Two of the studies in the present review employed email reminders. Email reminders have substantial limitations, often being overlooked or sent to junk folders. Agachi et al. (2023) reported emails as a form of reminder do not effectively increase physical activity uptake.

Müller, Khoo and Morris (Müller et al., 2016) demonstrated positive effects in a text messaging intervention, however, authors reported after the text message reminders ceased so did participation levels in PA. Conversely, studies included in this review such as Mansson et al. (2020) and Liu et al. (2021) utilised mobile apps which allow for more robust reminders and unlock more potential of mHealth by using customised workouts and linking with wearables and obtaining more data (Sohaib Aslam et al., 2020).

Delbaere et al. (2021) employed reminders built into a calendar within the app to promote PA, with promising results. However, these reminders were manually created by participants, which is likely to increase participant/user burden and does not really harness the power of digital technology (Seifert et al., 2020). Some studies used home visits as their method of reminding participants to take part in PA. For example, Taylor et al. (2019) reported that by week 12, only 54% of the desired PA dose was being completed by participants. Interestingly, the dose was set at 40 min increasing by 20 min every 2 weeks eventually reaching 120 min. It is possible this increase may have been too quick for some of the sample, which caused the high attrition. Future trials are needed over a longer period to gain a sense of appropriate increases in PA dose to maintain acceptable levels of adherence, but also achieve the desired physiological adaptations and disease risk reductions. It should be noted that the study in question only had a sample of 15, meaning that this % of participants completing the desired PA dose may mean the intervention is not scalable in the general population. Finally, smartphones may offer potential to enhance adherence. While tablets are typically used only at home, reliant on wireless local area networks (WLAN), smartphones are usually kept near to the body and allow for notifications to be delivered to participants in the moment (Wilson et al., 2022). Further to this point, smartphones can also be paired with wearable devices such as smartwatches which allow for ‘nudge theory’ to be applied. Nudge theory refers to subtly guiding decisions and behaviours (Cai, 2020). In this context, a wearable paired with a smartwatch can further enhance the potential for mHealth, as the wearable permits measurement of PA metrics (Mair et al., 2021) and allows for the delivery of just-in-time adaptive interventions [JITAIs (Mair et al., 2021)] to promote PA behaviours. An additional benefit is that the wearable can itself produce notifications or mirror those of the smartphone (Casado-Robles et al., 2022). Of course, owning a wearable requires resource and financial commitment and technical literacy, which may be perceived as a barrier to adoption, especially in older populations (Kim et al., 2023).

An important result of the present review is that all but one mHealth studies were conducted in participants’ natural environments. This enhances ecological validity, providing a realistic, authentic depiction of how interventions may perform in real-world settings [i.e., effectiveness rather than merely efficacy (Gartlehner et al., 2006)], facilitating replication (Messner et al., 2019). Despite the obvious potential and observed benefits of mHealth and eHealth research included in this review, there are cost implications of device ownership. This is a particular issue with tablet-based interventions as currently the latest Apple iPad retails at $1,265. This may be why eight out of the nine tablet interventions provided participants with a device and this must be considered a barrier to implementation at scale (Marcolino et al., 2018). However, as prices for tablet computers reduce, and digital literacy improves in older populations, the use of tablets may be beneficial for older adults with reduced dexterity and impaired vision as a larger screen may increase useability compared to a smartphone (Kim et al., 2022; Statistica, 2023).

#### Exergames

Of the included studies, exergaming was a popular approach (Karssemeijer et al., 2019; Szturm et al., 2011; Yang et al., 2020; Montero-Alía et al., 2019; Gschwind et al., 2015; Padala et al., 2017; Franco et al., 2012; Bieryla and Dold, 2013; Rosenberg et al., 2010), and the findings of these studies were mixed. Notably, interventions that spanned 3 weeks and 12 weeks (Montero-Alía et al., 2019; Franco et al., 2012) reported no meaningful improvements in balance. Conversely, the included Wii-Fit study by Roopchand-Martin et al. (2015) employed a 6-week intervention and reported improved balance which is in line with previous work by Nicholson et al. (2015). However, it should be noted this study had a sample size of 33, lacked a control group and did not complete a sample size calculation so such improvements in balance may be attributed to other regular daily activities and familiarity with the outcome measures. Exergames, like tablet interventions, require financial investment, with equipment costing $150-$250, making large-scale interventions potentially unfeasible (Klompstra et al., 2022).

#### Videoconferencing

Of the studies which used videoconferencing software (Schwartz et al., 2021; Li et al., 2022; Wong et al., 2005; Granet et al., 2023; Granet et al., 2022), those run remotely which utilised live sessions (Schwartz et al., 2021; Li et al., 2022; Wong et al., 2005; Granet et al., 2023; Granet et al., 2022) proved more effective than those which were pre-recorded (Granet et al., 2023; Granet et al., 2022), consistent with previous research by Klonova et al. (2022). One study which was held at a community centre resulted in lower attendance rates compared to remote studies, highlighting greater accessibility of entirely remote interventions, and how this may improve adherence (Jimison et al., 2013). It seems illogical to us to travel to a physical location to receive a remote intervention, and with improvements in technology over the past decade, this would unlikely occur in 2024 in real-world settings. Despite safety concerns in remote interventions (Gell et al., 2021), no adverse events were reported in the studies in the present review, as regular safety screenings and home visits were conducted.

#### DVDs and robotics

The studies using DVDs (Gothe et al., 2014; Yamada et al., 2011; Katrancha et al., 2015) reported positive results and this was in line with similar DVD interventions in older adults by McAuley et al. (2013), who reported balance improvements of 0.53 in SPPB rating in a 6-month DVD intervention. Higher attendances were observed in interventions held at senior community centres suggesting the need for direct guidance, as older adults may struggle with DVD functionality or adherence (Fanning et al., 2016). With the rise of apps such as Apple Fitness+, it is possible to implement interventions similar to those that have used DVDs to mobile apps using elements such as home workouts through inbuilt streaming services accessed via a smartphone, smart TV, laptop or tablet rather than a DVD player, in keeping with technological advancements (Chen et al., 2023).

Robotics studies reported improvements in gait and balance improvements (Lee et al., 2022; Ozaki et al., 2017). However, the benefits of mHealth far outstrip the time and cost burden of robotic interventions. We therefore believe research should pursue mHealth instead, certainly in larger scale interventions with ‘healthy’ older adults (Fernandez-Garcia et al., 2021). As discussed, the rise of fitness streaming services offers an avenue to streamline these successful methodologies into an mHealth approach.

### Reported outcomes (feasibility, usability, and efficacy)

#### Feasibility

The third objective was to outline outcomes reported in included studies (usability, feasibility, and efficacy). Most studies found digital interventions feasible (Bowen et al., 2022; Liu et al., 2021; Karssemeijer et al., 2019; Yang et al., 2020; Gschwind et al., 2015; Padala et al., 2017; Schwartz et al., 2021; Taylor et al., 2019; Mansson et al., 2020; Silveira et al., 2013; Li et al., 2022; Wong et al., 2005; Granet et al., 2023; Jansons et al., 2021; Granet et al., 2022; Daly et al., 2021; Rosenberg et al., 2010; van Het Reve et al., 2014; Geraedts et al., 2017) for older adults, though adherence was less clear, with just over half meeting their own criteria. High adherence was most common in smartphone interventions (Bowen et al., 2022; Mansson et al., 2020; Shake et al., 2018) (95%), aligning with Alasfour and Almarwani (Alasfour and Almarwani, 2020), who attributed increased adherence to the attractive and motivational features of the smartphone app. This emphasises the potential of well-designed mHealth applications to sustain adherence (Tajudeen et al., 2021). In the context of the present study the adherence rates are high in comparison to other intervention delivery types, for example, one of the included interventions which used the Wii Fit (Rosenberg et al., 2010) registered an adherence rate of 84% in a 12-week intervention including two weekly sessions which were 30 min in duration. A tablet intervention conducted over 2 weeks with 10 PA sessions lasting approximately 1 h in duration also reported good adherence to their PA intervention (73%) (Silveira et al., 2013). It is also important to note, both the studies had a higher sample size than the mHealth study, but still less adherence in terms of actual number of sessions attended indicating that boarder scale mHealth studies may have even more potential for increased adherence. Exergame interventions also had high adherence. Anderson-Hanley (Anderson-Hanley et al., 2012) reported 80% adherence in their exergame intervention, and Pacheco et al. found that all studies using Wii Fit had adherence levels above 90%, with none below 80%. Exergames engage older adults through enjoyable PA, likely explaining higher adherence (Pacheco et al., 2020). Yet, most studies reported herein were of short duration (up to 12 weeks) and Höchsmann et al. (2019) suggested greater long-term adherence for smartphone interventions due to personalising the user experience and goal setting, an area where exergames often fall short may be plausible.

The highest rates of attrition (∼17%) were found in two studies (Mansson et al., 2020; Silveira et al., 2013) which used both mHealth and eHealth approaches (smartphones and tablets) respectively. It is important to note, one intervention lasted 4 months, which is a particularly long intervention time in comparison to the other study and may have influenced the level of attrition observed. However, it is important to note that this length of time gives a greater indication of real-world adherence and is a crucial consideration for the sustainability and lasting impact of the intervention. Previous work by Devereux-Fitzgerald et al. (2016), found long interventions in older adults often cause boredom or too much cognitive load resulting in high attrition rates. One of the included studies with a relatively high attrition rate attributed this to connectivity issues. This is in line with the RCT completed by Baez et al. (2017) which had an attrition rate of 8%. The higher rate of participant drop out was attributed to poor internet connection which could not be solved. Thus, it is key that interventions consider including offline functionality within their technology to allow participants to benefit during times where connection may drop off (Sen et al., 2022). Future mHealth and eHealth interventions should consider internet connectivity issues and methods to overcome them to maintain participation. This could be implemented by minimising data requirements, including offline content, or including lower data requirements (e.g., alternative text instructions when video playback is unavailable). Therefore, we suggest a focus on mHealth studies with key considerations for connection and cognitive load, well designed mobile apps with offline functionality would be able to surpass the barriers faced by studies in the present review.

The highest recruitment rates were seen in interventions employing wearable devices combined with smartphone apps (93%). In previous studies, wearable devices have shown good recruitment and retention rates in older adults (Ehn et al., 2018). However, a previous focus group (Kononova et al., 2019) reported older adults found it difficult to remember to wear the activity tracker. Conversely, Brickwood et al. (2020) managed to recruit 365 older adults to their RCT. This study highlighted the live data tracking of participants’ PA was a particular strength, as most participants were interested by these insights. This speaks to work from our own laboratory, whereby we completed a JITAI to maintain PA during the COVID-19 lockdown and a large proportion of participants would navigate to the wearable’s companion app for deeper insights into their PA completion (Mair et al., 2021). This was surprising to us as we intended to limit participant burden, but in fact participants wanted the information, despite the burden.

With regards to retention, high rates were found in videoconferencing interventions (94%). Despite this positive finding, the scalability of such eHealth interventions is limited by the time constraints on calls and the maximum number of participants that can participate in videoconferencing (Klonova et al., 2022). We therefore suggest the positive aspects of these intervention types such as the social motivation on live PA calls be channelled into larger studies taking an mHealth route.

#### Usability

High usability was reported in exergame and robotics interventions respectively as per study feedback questionnaires. Participants highlighted that over time they were able build up technical competence in using the equipment (Rosenberg et al., 2010), this is in line with a previous review that stated in most studies older adults rated exergames as highly usable (Nawaz et al., 2016). It should be noted that both interventions reported in this review took place in laboratory setting with researcher support. We argue this limits authenticity, scalability, and reach, reducing ecological validity and thus rendering this type of PA support unsuitable for population-level implementation.

High SUS scores were observed in mHealth interventions included in the review. This is in line with previous smartphone interventions by Kim et al. (2020) who had a post intervention SUS score of 72 in their cohort. For context, the SUS contains 10 items scored from one to five on a Likert scale with scores above 68 considered above average (Lewis, 2018). Similarly, work by Perotti et al. (2024) also found high SUS scores in an online intervention employing smartphones and tablets. The study by Lee and Ryu (Lee and Ryu, 2023) highlighted these interventions are particularly usable as a training function can be built into the app, which further supports older adults in getting the best out of the intervention. However, one eHealth study which dropped below average SUS score (61) was a web-based tablet intervention. This highlights the need for apps and websites within interventions to be better designed in line with older adults needs and future research in mHealth/eHealth interventions should build ‘how to videos’ to further improve usability scores (Darley et al., 2022). Further to this, we suggest that research should steer towards using mHealth interventions to their full potential by building apps rather than employing a single browser on a small screen.

#### Efficacy

Of studies reporting efficacy, concerningly only two (Liu et al., 2021; Yamada et al., 2011) completed an *a priori* sample size calculation, limiting confidence in results (Gupta et al., 2021). Efficacy was observed in physical performance outcomes across a range of videoconferencing interventions. This is in line with previous research by Wu and Keyes (Wu and Keyes, 2006) which demonstrated the potential for videoconferencing interventions to improve a range of balance and functional parameters in older adults, noting participants were highly satisfied with the interventions format. Similarly, positive effects were also found for the same outcomes in those studies in the review employing a tablet intervention. This is also in line with previous literature by Nikitina et al. (2018). Despite this, in one of the included videoconferencing studies by Granet et al. (2023), only the live group improved. Therefore, despite positive findings in both digital intervention types, tablet approaches offer the opportunity for further, more in-depth coaching and scalability improving the intervention outcomes (Adebayo et al., 2023).

The efficacy of exergame interventions for improving balance and physical fitness was heterogeneous, with notable shortcomings. This contrasts with Hernandez-Martinez et al. (2024)’s meta-analysis, which found exergames effective for enhancing balance in older adults across 10 studies. However, the interventions in their meta-analysis spanned up to 20 weeks, while some in the current review lasted only 3 weeks (Franco et al., 2012). Previous literature (Malbut et al., 2002) has reported 12 weeks as a minimum duration for improvements in VO_2max_ in older adults, which may indicate that studies in the current review may have been too short in duration to produce desired effects, indicating a need for research to consider longer interventions (Fitzgerald et al., 1997).

Studies reporting on muscular adaptations generally showed favourable effects. Improvements were seen in videoconferencing interventions (Wong et al., 2005; Granet et al., 2023; Granet et al., 2022), in line with previous research by Edna Mayela et al. (2023) who reported increased muscular strength and endurance in older adults in a Zoom delivered PA RCT intervention lasting up to 36 weeks with two to five sessions delivered weekly. To the best of the authors knowledge there are no mHealth interventions targeting muscular adaptations in the literature. This is a concerning and notable finding, given the considerable economic burden of sarcopenia (Granic et al., 2019), a progressive skeletal muscle disorder characterised by reduced skeletal muscle quantity and function. Sarcopenia is associated with a range of negative health outcomes including frailty, falls, reduced quality of life and mortality (Granic et al., 2019; Cruz-Jentoft et al., 2018). The estimated current cost of sarcopenia is ∼£3 billion per year in the United Kingdom (Langhammer et al., 2018). Older adults exhibit high levels of physical inactivity or sedentariness (Graham et al., 2024), but even fewer complete the recommended muscle strengthening exercise volume (Hayes et al., 2020). Therefore, given the need for muscle strengthening interventions in older adults, we would have expected more mHealth interventions targeting muscle strength.

In terms of efficacy in increasing PA, success was found in those interventions who employed a tablet and wearable device intervention. While exergame interventions struggled to increase PA, levels post interventions. This is in line with previous research which has found mHealth and wearable interventions efficacious in improving PA levels in older adults (Schmidt et al., 2022). Notably, the tablet and wearable interventions were up to 50% shorter than those using exergames. These findings suggest that tablet and wearable devices have more potential for increasing PA in older adults than exergames. This may be due to the unique personalisation features in mHealth interventions which may not be replicable in exergame settings. This allows older adults to set their own goals around PA and in turn increasing their motivation (Thornton et al., 2017).

Further studies utilising videoconferencing software (Wong et al., 2005) and tablets (Delbaere et al., 2021) reported positive effects via EQ-5D and SF-36 scores, these are questionnaires which measure overall sense of health and wellbeing. These findings are in line with previous research showing similar effects in these intervention types (Lim et al., 2022). As well as being efficacious at improving sense of health and wellbeing, studies in the included review also helped improve physical health measures such as body composition (Bowen et al., 2022). These findings highlight the potential for overall health and wellbeing effects in long term mHealth interventions underlining the need for further developments (Garnett et al., 2021). Overall, the included studies demonstrated efficacy across a wide range of digital interventions. Notably, the significant scalability of mHealth interventions presents enormous potential. Therefore, integrating the effects observed in eHealth and various PA protocols into future mHealth studies could ensure optimal results.

### Understand user perspectives

Higher participant satisfaction levels were observed in smartphone and videoconferencing interventions (100% and 97% respectively). These findings agree with previous literature by Mair et al. (2021) and Cohen-Mansfield et al. (2021) in which consistent high user satisfaction was reported. Effective eHealth features, such as live coaching and social interaction seen in videoconferencing (Fyfe et al., 2022) could be adapted into mHealth interventions but would result in decreased personalisation or reach because one ‘coach’ cannot personalise feedback for hundreds of thousands of users.

In the current review, participant feedback underscored that usability was less clear in tablet-based interventions (Alley et al., 2023), particularly concerning the in-built PA plan features within the apps. Notably, the study that identified this (Alley et al., 2023) was a larger-scale intervention (sample size ≈120). This finding is significant, as previous research by Soto-Bagaria et al. (2023) also highlighted usability challenges with apps in larger-scale interventions. Given that even effective interventions do not work for all participants (Edlind et al., 2018), it may be pragmatic to accept lower usability for increased reach or sample size. By this we mean it may be preferred if half of ten million participants experience a positive effect of an intervention despite faults, rather than 100% of 100 participants experiencing a positive effect of the better-designed intervention.

When measuring usability of their intervention, only one included study used a validated questionnaire or survey. Granet et al. (2023) employed the Motivation Scale towards PA in a Health Context (MSPAHC), which is specifically designed to measure motivation for PA rather than the effectiveness of digital interventions. This limitation highlights a significant gap in the current research. It suggests a pressing need for future studies to incorporate instruments like the mHealth App Usability Questionnaire (MAUQ) to properly assess usability, as recommended by Zhou et al. (2019) and it is therefore, difficult to generalise questionnaire findings in the current review due to their divergent domains. A promising finding was 100% usability in included mHealth studies (Bowen et al., 2022; Mansson et al., 2020; Shake et al., 2018), indicating strong potential for future interventions.

### Recommendations for advancement in the investigative area

This review found no studies examining muscle function via a smartphone app. In this regard, only five of the included studies (Lee et al., 2022; Szturm et al., 2011; Wong et al., 2005; Granet et al., 2023; van Het Reve et al., 2014) measured muscular outcomes with four out of five observing improvements (Lee et al., 2022; Wong et al., 2005; Granet et al., 2023; van Het Reve et al., 2014), demonstrating the potential for remote muscle strengthening interventions. Thus, the primary recommendation from this review is to increase mHealth studies considering muscle strengthening in older adults. mHealth offers advantages over eHealth, such as portability, enhanced communication, and scalability (Bergevi et al., 2022). Since mobile internet usage surpassed desktop in 2016, leveraging mHealth is crucial (Stat Counter - Global Stats, 2016). A more specific recommendation is the utilisation of mobile applications as the primary mHealth intervention type. Using apps allows for a new level of accessibility and participant convenience which cannot be found in eHealth types (Samari et al., 2024), further to this, the use of push notifications can act as timely reminders to participants to stay motivated and visualise their own progress (Hernández-Reyes et al., 2020). With the increase in smartphone ownership and the benefits underlined in using this approach mHealth seems a suitable and scalable way forward for digital exercise interventions to reach their full potential (Domin et al., 2021). mHealth is a cost effective and scalable solution for digital exercise interventions (Wunsch et al., 2024). Much of the included studies used eHealth approaches such as exergames, which as discussed have financial barriers for researcher, participant or both (Warlo et al., 2024). Furthermore, this technology is often not readily available in older adults’ homes, unlike smartphones.

Addressing muscle strengthening is vital because few older adults meet the PA guidelines for muscle strengthening activities (Strain et al., 2016), risking sarcopenia, reduced stability and mobility, decreased bone density, and chronic diseases (Lu et al., 2023). Although muscle-strengthening activities are harder to measure than aerobic activities, researchers and professionals should not avoid muscle strengthening interventions. The second recommendation is to learn from successful eHealth strategies in terms of usability, feasibility, and acceptability, and adapt them for mHealth, benefiting from its time-efficient approach (Sohaib Aslam et al., 2020). Thirdly, only seven (∼20%) included studies (Gothe et al., 2014; Karssemeijer et al., 2019; Montero-Alía et al., 2019; Gschwind et al., 2015; Delbaere et al., 2021; Alley et al., 2023; Shake et al., 2018) had a sample size over 100, and only six had interventions longer than 3 months. Long-term, large-scale studies are needed despite their cost and time commitment, as they allow participants to familiarise themselves with new technology and help researchers identify and address attrition (Vaportzis et al., 2017). It is also hoped further research can implement behavioural change in order for participants to continue their new exercising habits in turn further reducing long term pressure on the National Health Service (NHS). Finally, further studies are necessary to evaluate the feasibility, usability, and efficacy of mHealth muscle-strengthening approaches, to ensure best practices.

### Strengths and limitations

Within this review there are several strengths and limitations that must be considered. Firstly, the included studies used a vast range of digital exercise interventions. Studies were carried out across a range of settings utilising different intervention types, modes of exercise, difficulty of exercise, and a range of different participants at differing levels of abilities. This heterogeneity made direct comparisons between interventions challenging which may be a limitation of this review. That said, our *a priori* aim was to catch a broad range of interventions and identify strengths and limitations of each area, so this could also be perceived as a strength of the current review. It should be noted that 63% of included studies involved older adults between 60 and 75 and so it may be the case that findings in this age group may not manifest in older age groups (80+), further research is needed in this age group to clarify. Within the included studies there was a focus on older adults who were inactive and as such, there may be recruitment bias and results may not extend to active older adults. Further to this, a small minority included older adults with degenerative diseases and as such further research is needed to confirm findings in those with comorbidities. This review may have been subject to publication bias as the vast majority of included studies had positive findings in either feasibility, usability or efficacy, as studies with positive findings are more likely to be published, this may lead to an overestimation of the effectiveness of these intervention types in line with the outcome measures. Furthermore, as stipulated in Supplementary Table 1, much of the research took place in high income countries where there is likely a good standard of digital literacy. This limits the findings applicability to developing nations populations and therefore, further investigations in these settings are needed to establish intervention suitability. Furthermore, studies involved participants from different sexes and further research is needed to observe the impact this has on digital exercise intervention implementation. Lastly, the inclusion criteria stipulated studies must be published in English and therefore, it is possible robust interventions have been missed that have been published in other languages.

## Conclusion and practical recommendations

Overall, there is an evident absence of mHealth approaches in the literature, with 20 of the included studies using eHealth. Most mHealth studies involved tablet interventions, highlighting a need for more smartphone application studies. We do expect that mHealth studies will proliferate over the coming years, with the increasing ease of app development such as “no-code” and R packages like Shiny now making app development more accessible. Additionally, there was a lack of muscle-strengthening interventions via smartphone apps. We hope the increasing ease of app development will facilitate increased research interest in muscle strengthening approaches, despite the challenge of measuring muscle function. Before long-term RCTs which are necessary to test efficacy or effectiveness, feasibility, usability, and efficacy, studies are required to ensure the greatest chance of future behaviour change and efficacy. This review provides a comprehensive resource for future research and indicates older adults are comfortable using digital interventions, including smartphones. mHealth could offer a cost-effective, scalable, and sustainable means to target muscle strengthening. In conclusion, digital interventions are generally feasible, usable, and effective in older adults, and this review’s findings can inform future work.

## Data Availability

The original contributions presented in the study are included in the article/Supplementary Material, further inquiries can be directed to the corresponding author.
