# Supplementary material for: A scoping review of the feasibility, usability, and efficacy of digital interventions in older adults concerning physical activity and/or exercise
Source: Front Aging. 2025 Apr 11;6:1516481. doi: 10.3389/fragi.2025.1516481 (PMC12021916; doi:10.3389/fragi.2025.1516481)
Supplement: Supplementary file 1 [file Table2.docx]

| **Study** | **Study Duration** | **Intervention** | **Participant N (Mean ± SD Age)** | **Sex** | **Outcome Measure** | **% Increase/Decrease from Baseline** | **Sig.** |
| --- | --- | --- | --- | --- | --- | --- | --- |
| Granet et al.^52^ | 12 weeks | Videoconferencing intervention conducted via Zoom. Mixture of aerobic, functional and resistance training 3x 1-hour sessions per week. | 83 participants (70 ±5.1) | M = 16  F = 67 | SPPB  10STS  30-sec CST | Live group = 5%  Recorded group = -1%  Live group = 60%  Recorded group = 10%  Live group = 33%  Recorded group = 22% | Yes  No  Yes  No  Yes  No |
| Van Het Reve et al.^61^ | 12 weeks | Tablet intervention conducted via the ‘ActiveLifestyle’ app. Mixture of 2x resistance and 5x balance training sessions per week. | 44 participants (75 ± 8.6) | M = 16  F = 28 | SPPB | Brochure group = 12%  Social group = 23%  Individual group = 11% | Yes  Yes  Yes |
| Bieryla and Dold.^55^ | 3 weeks | Exergame intervention conducted via the Wii Fit. Mixture of 3x sessions of balance and aerobic sessions per week. | 12 participants (82 ± 5.5) | M = 2  F = 10 | BBS  FAB  FRT  TUG | Experimental = 6%  Control = 5.8%  Experimental = 5%  Control = 3%  Experimental = -3%  Control = 0%  Experimental = -6%  Control = -12.5% | Yes  No  No  No  No  No  No  No |
| Karssmeijer et al.^33^ | 12 weeks | Exergame intervention conducted via an exercise bike connected to a screen. Aerobic training 3x per week for 30-50 minutes at 65-75% HR reserve. | 115 participants (79 ± 6.9) | M = 62  F = 53 | TUG  5TSTS  10 metre walk test  SPPB | Experimental = - 2%  Control = 4%  Experimental = -8%  Control = 7%  Experimental = - 4%  Control = -8%  Experimental = 4%  Control = 2% | No  No  No  No  No  No  No  No |
| Lee et al.^34^ | 12 weeks | Robotics intervention delivered via hip exoskeleton. Mixture of weekly walking and resistance activity dependent on study group. | 60 participants (75 ± 4.1) | M = 30  F = 30 | 10 metre walk test  SPPB  BBS  FRT  TUG | Largest increase reported per test by group:  Group D = 7%  Group D = 7%  Group D = 8%  Group D = 18%  Group B = -21% | Yes  Yes  Yes  Yes |
| Szturm et al.^35^ | 8 weeks | Exergame intervention. Strength training completed 2x per week for 45 minutes. | 30 participants (81 ± 6.5) | M = 11  F = 19 | BBS  TUG | Experimental = 21%  Control = 21%  Experimental = 20%  Control = 51% | Yes  Yes  Yes  Yes |
| Katrancha et al.^56^ | 12 weeks | DVD intervention. Aerobic and balance training completed 3x per week for 45 minutes. | 32 participants (73 ± 8.6) | M = 3  F = 29 | COB measured via the Wii Fit balance board | Eyes open right = 3%  Eyes open left = -3% | Yes  Yes |
| Yang et al.^36^ | 5 weeks | Exergame intervention. Balance training completed 2x per week for 45 minutes. | 20 participants (68) | M = 2  F = 18 | 30 Sec-CST  TUG  FRT  OLST | Experimental = 38%  Control = 21%  Experimental = -14%  Control = -13%  Experimental = 16%  Control = 18%  Experimental = 146%  Control = 17% | Yes  Yes  Yes  No  Yes  Yes  Yes  Yes |
| Shake et al.^53^ | 10 weeks | Tablet intervention delivered via the ‘Bingocize’ app. Mixture of aerobic, balance and resistance training completed 2x per week for 1 hour. | 105 participants (73 ± 7.8) | M =15  F = 90 | 30-sec CST  4m walk test | Experimental = - 17%  Control = -5%  Experimental = 8%  Control = 6% | Yes  No  No  No |
| Yamada et al.^37^ | 24 weeks | DVD intervention. Resistance and agility training completed 2x per week for 20 minutes. | 84 participants (83±6.1) | M = 19  F = 65 | TUG  5TSTS | Experimental = 2%  Control = -2%  Experimental = -2%  Control = - 1% | No  No  No  No |
| Montero-Alia et al.^38^ | 12 weeks | Exergame intervention delivered via the Wii Fit. Balance training completed 2x per week for 30 minutes. | 977 participants (75) | M = 400  F = 577 | Tinetti’s Balance Test | Experimental = 0%  Control = 2% | No  No |
| Roopchand-Martin et al.^57^ | 6 weeks | Exergame intervention delivered via the Wii Fit. | 33 participants (70 ± 6.7) | M = 7  F = 26 | BBS | Single group pre-test/post-test = 3% | Yes |
| Wong et al.^48^ | 12 weeks | Videoconferencing intervention. Resistance and functional training completed 3x per week. | 20 participants (75 ± 7) | M = 2  F = 18 | TUG  BBS | Single group pre-test/post-test = -21%  Single group pre-test/post-test = 12% | Yes  Yes |
| Franco et al.^54^ | 3 weeks | Exergame intervention delivered via the Wii Fit. Balance training completed 2x per week 10-15 minutes. | 32 participants (78 ± 6) | M = 7  F = 25 | BBS  Tinetti’s balance test | Wii Fit = 7%  MOB = 7%  Control = 2%  Wii Fit = 3%  MOB = 5%  Control = 4% | No  No  No  No  No  No |
| Granet et al.^49^ | 12 weeks | Videoconferencing intervention delivered via Zoom. Mixture of aerobic and resistance training completed 3x per week for 1 hour. | 46 participants (60) | M = 13  F = 33 | SPPB  TUG  30-sec CST | Live-recorded-live group = 7%  Recorded-live-recorded group = 2%  Live-recorded-live group = - 8%  Recorded-live-recorded group = - 8%  Live-recorded-live group = 31%  Recorded-live-recorded group = 30% | No  No  No  No  Yes  Yes |
| Gswhind et al.^39^ | 12 weeks | Exergame intervention delivered via Microsoft Kinect. Balance training completed 3x per week for 40 minutes and resistance training completed 3x per week for 15-20 minutes. | 153 participants (75 ± 6.5) | M = 60  F = 93 | SPPB  TUG | Experimental = 8%  Control = 7%  Experimental = - 2%  Control = - 10% | No  No  No  No |
| Padala et al.^40^ | 8 weeks | Exergame intervention delivered via the Wii Fit. Aerobic and resistance training completed 3x per week for 45 minutes. | 30 participants (68 ± 6.7) | M = 26  F = 4 | BBS | Experimental = 8%  Control = 0% | Yes  No |
| Jansons et al.^50^ | 12 weeks | Voice activation intervention delivered via Amazon Alexa. Resistance training completed in ‘snacks’ at 2x per day, 3x per day and 4x per progressing in 4-week stages. | 15 participants (70 ± 4) | M = 6  F = 9 | 30-sec CST | Single group pre-test/post-test = 10% | No |
| Delbaere et al.^41^ | 2 years | Tablet intervention delivered via the ‘StandingTall’ app. Balance training completed 2 hours per week minimum. | 503 participants (77 ± 5.5) | M = 164  F = 339 | TUG  5TSTS  10m walk  SPPB | Experimental = -3%  Control = 0%  Experimental = - 11%  Control = -7%  Experimental = - 2%  Control = - 2%  Experimental = 0%  Control = 0% | No  No  No  No  No  No  No  No |
| Ozaki et al.^60^ | 12 weeks | Robotics intervention delivered via the ‘BEAR’ system. Resistance and balance training completed 2x per week. | 27 participants (73 ± 6) | M = 7  F = 20 | Gait speed  TUG  FRT | Experimental = 4%  Control = 2%  Experimental = - 7%  Control = -3%  Experimental = 10%  Control = 1% | Yes  No  Yes  No  Yes  No |
