# Supplementary material for: A scoping review of the feasibility, usability, and efficacy of digital interventions in older adults concerning physical activity and/or exercise
Source: Front Aging. 2025 Apr 11;6:1516481. doi: 10.3389/fragi.2025.1516481 (PMC12021916; doi:10.3389/fragi.2025.1516481)
Supplement: Supplementary file 2 [file Table3.docx]

**Database Search Protocol**

Databases Searched:

1. MEDLINE
2. CINAHL Ultimate
3. Scopus
4. Cochrane Central Register of Controlled Trials (CENTRAL)

**Search Terms**

**1.0 MEDLINE**

| **Type** | **Terms** | **Searches** |
| --- | --- | --- |
| **Population** | Mesh terms | Independent Living or Housing for the Elderly and Aged, 80 and over" or Frail Elderly or Aged |
|  | Free-text | (communit* N3 dwell* or residen*) AND (elderly or geriatric or age* or aging) |
| **Intervention** | Mesh terms | Text Messaging or Smartphone or exp Cell Phone or Mobile Applications or Social Media or exp Telemedicine or Internet-Based Intervention or exp Wearable Electronic Devices or exp Accelerometery or exp Computer Simulation or exp Video Games or Gamification or exp Artificial Intelligence or exp Machine Learning |
|  | Free-text | (text* or SMS or "mobile device" or "mobile phone” or “mobile health” or mHealth or eHealth or internet-based or web-based or DVD-based or (wearable N3 (devic* OR technol*)) or computer or “computer assisted” or (serious N3 game*) or tablet or "artificial intelligence" or AI) |

| # | Searches | **Results** |
| --- | --- | --- |
| 1 | TI ( (communit* N3 (dwell* or residen*)) AND (elderly or geriatric or age* or aging ) ) OR AB ( (communit* N3 (dwell* or residen*)) AND (elderly or geriatric or age* or aging ) ) | 36,788 |
| 2 | (MH "Independent Living") OR (MH "Housing for the Elderly") | 13,440 |
| 3 | (MH "Aged, 80 and over") OR (MH "Frail Elderly") OR (MH "Aged") | 3,474,321 |
| 4 | 2 AND 3 | 11,718 |
| 5 | (MH “Text Messaging”) or (MH ”Smartphone”) or (MH ”Cell Phone+”) or (MH ”Mobile Applications”) or (MH ”Social Media”) or (MH ”Telemedicine+”) or (MH ”Internet-Based Intervention”) or (MH ”Wearable Electronic Devices+”) or (MH ”Accelerometery+”) or (MH ”Computer Simulation+”) or (MH ”Video Games+”) or (MH ”Gamification”) or (MH ”Artificial Intelligence+”) or (MH ”Machine Learning+”) | 579,048 |
| 6 | TI ( (text* or SMS or "mobile device" or "mobile phone” or “mobile health” or mHealth or eHealth or internet-based or web-based or DVD-based or (wearable N3 (devic* OR technol*)) or computer or “computer assisted” or (serious N3 game*) or tablet or "artificial intelligence" or AI) ) OR AB ( (text* or SMS or "mobile device" or "mobile phone” or “mobile health” or mHealth or eHealth or internet-based or web-based or DVD-based or (wearable N3 (devic* OR technol*)) or computer or “computer assisted” or (serious N3 game*) or tablet or "artificial intelligence" or AI) ) | 681,043 |
| 7 | 5 OR 6 | 1,159,071 |
| 8 | 1 OR 4 | 40,381 |
| 9 | 7 AND 8 | 1,568 |
| 10 | Limiters: Publication Date: 1995-2024; English Language; Human | 1,397 |

**2.0 CINAHL Ultimate**

| **Type** | **Terms** | **Searches** |
| --- | --- | --- |
| **Population** | Mesh terms | exp Community Living or Housing For Older Persons and exp Aged, 80 and over" or Frail Elderly or Aged |
|  | Free-text | (communit* N3 (dwell* or residen*) AND (elderly or geriatric or age* or aging) |
| **Intervention** | Mesh terms | exp Computer Simulation or exp Artificial Intelligence or exp Video Games or exp Virtual Reality or Text Messaging or Cellular Phone or Smartphone or exp Telehealth or Telemedicine or Remote Consultation or Telerehabilitation or Telenursing or exp Internet or Instant Messaging or exp Accelerometery |
|  | Free-text | (text* or SMS or "mobile device" or "mobile phone” or “mobile health” or mHealth or eHealth or internet-based or web-based or DVD-based or (wearable N3 (devic* OR technol*)) or computer or “computer assisted” or (serious N3 game*) or tablet or "artificial intelligence" or AI) |

| **#** | **Searches** | **Results** |
| --- | --- | --- |
| 1 | TI ( (communit* N3 (dwell* or residen*)) AND (elderly or geriatric or age* or aging ) ) OR AB ( (communit* N3 (dwell* or residen*)) AND (elderly or geriatric or age* or aging ) ) | 21,848 |
| 2 | (MH “Community Living+”) OR (MH "Housing for Older Persons”) | 29,323 |
| 3 | (MH "Aged") OR (MH "Aged, 80 and Over+") OR (MH "Frail Elderly") | 962,343 |
| 4 | 2 AND 3 | 20,036 |
| 5 | (MH "Computer Simulation") OR (MH "Artificial Intelligence+") OR (MH "Video Games+") OR (MH "Virtual Reality+") OR (MH "Text Messaging") OR (MH "Cellular Phone") OR (MH "Smartphone") OR (MH "Telehealth+") OR (MH "Telemedicine") OR (MH "Remote Consultation") OR (MH "Telerehabilitation") OR (MH "Telenursing") OR (MH "Internet+") OR (MH "Instant Messaging") OR (MH "Accelerometery+") | 271,258 |
| 6 | TI ( (text* or SMS or "mobile device" or "mobile phone” or “mobile health” or mHealth or eHealth or internet-based or web-based or DVD-based or (wearable N3 (devic* OR technol*)) or computer or “computer assisted” or (serious N3 game*) or tablet or "artificial intelligence" or AI) ) OR AB ( (text* or SMS or "mobile device" or "mobile phone” or “mobile health” or mHealth or eHealth or internet-based or web-based or DVD-based or (wearable N3 (devic* OR technol*)) or computer or “computer assisted” or (serious N3 game*) or tablet or "artificial intelligence" or AI) ) | 169,446 |
| 7 | 5 OR 6 | 401,112 |
| 8 | 1 OR 4 | 32,557 |
| 9 | 7 AND 8 | 1,662 |
| 10 | Limiters: Publication Date: 1995-2024; English Language; Human | 1,330 |

**3.0 Scopus**

| **#** | **Search Terms** | **Results** |
| --- | --- | --- |
| 1 | ( TITLE-ABS-KEY ( elderly OR geriatric OR age* OR aging ) ) AND ( TITLE-ABS-KEY ( communit* W/3 ( dwell* OR residen* ) ) )AND ( TITLE-ABS-KEY ( text* OR sms OR "mobile device" OR "mobile phone" OR "mobile health" OR mhealth OR ehealth ORinternet-based OR web-based OR dvd-based OR ( wearable W/3 ( devic* OR technol* ) ) OR computer OR "computer-assisted"OR ( serious W/3 game* ) OR tablet OR "artificial intelligence" OR ai ) ) AND ( LIMIT-TO ( LANGUAGE , "english" ) ) AND ( LIMIT-TO ( EXACTKEYWORD , "human" ) OR LIMIT-TO ( EXACTKEYWORD , "humans" ) ) | 1,477 |

**4.0 Cochrane Central Register of Controlled Trials** (**CENTRAL)**

| **ID** | **Search** | **Results** |
| --- | --- | --- |
| #1 | MeSH descriptor: [Independent Living] this term only | 985 |
| #2 | MeSH descriptor: [Housing for the Elderly] this term only | 42 |
| #3 | #1 OR #2 | 1024 |
| #4 | MeSH descriptor: [Aged] explode all trees | 256396 |
| #5 | #3 AND #4 | 926 |
| #6 | ((elderly or geriatric or age* or aging) AND (communit* near/2 (dwell* or residen*))):ti,ab,kw (Word variations have been searched) | 5330 |
| #7 | #5 OR #6 | 5549 |
| #8 | MeSH descriptor: [Text Messaging] this term only | 1522 |
| #9 | MeSH descriptor: [Smartphone] this term only | 1051 |
| #10 | MeSH descriptor: [Cell Phone] this term only | 939 |
| #11 | MeSH descriptor: [Mobile Applications] this term only | 1633 |
| #12 | MeSH descriptor: [Social Media] this term only | 598 |
| #13 | MeSH descriptor: [Telemedicine] this term only | 3631 |
| #14 | MeSH descriptor: [Internet-Based Intervention] this term only | 575 |
| #15 | MeSH descriptor: [Wearable Electronic Devices] explode all trees | 850 |
| #16 | MeSH descriptor: [Accelerometry] explode all trees | 1330 |
| #17 | MeSH descriptor: [Computer Simulation] explode all trees | 3528 |
| #18 | MeSH descriptor: [Video Games] explode all trees | 1062 |
| #19 | MeSH descriptor: [Gamification] this term only | 28 |
| #20 | MeSH descriptor: [Artificial Intelligence] explode all trees | 2986 |
| #21 | MeSH descriptor: [Machine Learning] explode all trees | 941 |
| #22 | ((text* or SMS or "mobile device" or "mobile phone" or "mobile health" or mHealth or eHealth or internet-based or web-based or DVD-based or (wearable near/3 (devic* OR technol*)) or computer or "computer assisted" or (serious near/3 game*) or tablet or "artificial intelligence" or AI)):ti,ab,kw | 129243 |
| #23 | {or #8-#22} | 137398 |
| #24 | #7 and #23 | 574 |
